# Scarlet Fever

**Published:** 1886-05

**Authors:** 


### SCARLET FEVER.

There is more affected by good nursing, than by medicine in nearly all cases of Scarlet Fever. It is essential that the nurse be one who has had experience of or who has been a close observer of cases of this kind. In fact, it is true of all fevers, that good and intelligent nursing is the best security against their fatal termination. Nor is it advisable to change nurses if it can be avoided. The nurse should have all the details of the case under her own charge, she should not be heavily clothed ; but should wear as little as possible should change it every day and have it washed or thoroughly aired. Neither should she make a dressing room of the sick room, but should make her toilet and keep her dresses in an adjourning room, where there is any possibility of doing so. Towels, blankets, sheets, curtains everything that in any way comes in contact with the patient should be kept entirely separate until they are thoroughly boiled and aired. This is essential to the health of the household, often to the whole neighborhood.

The room should be kept thoroughly ventilated, either by keeping open a window in the adjoining room or by some arrangement attached to the window of the sick room which will allow the ingress and egress of air without a draft; its temperature should be kept at about 60 deg., and regulated by a thermometer. If the room receives its heat from a furnace, the hot air should be made to pass over a pail of water containing either Labarraque's solution of Platt's Chlorides, and a towel with one end dipped in such a solution should be tacked over the register. If there be a stove, or, better than all, an open grate, these solutions can be placed near by, so as to be readily evaporated and distributed throughout the room.

The chamber should always contain some such solution in which to receive the excreta. A small quantity of urine should daily be collected in a clean vessel for the doctor's examination. It is usual to anoint the child with some greasy substance; this allays the intense itching or prickling, which is most annoying; it softens the skin, which is inflammed and swollen; it depresses the fever to a certain extent, and it serves to collect the scales, which, if shed, serve as carriers of contagion, and which are usually shed in flakes. The child should have its mouth washed once or twice daily, as also other parts of its body, for purposes of cleanliness, and the water used can contain either Labarrque's solution or vinegar Listerine, and possibly the doctor will order the frequent use of the hand-spray, such as is employed with cologne, using some good disinfectant for the throat in these cases.

Scarlatina, as far as we know at the present time, only comes from previous cases of the disease. Cleanliness not only lessens the danger of serious complications which are often fatal, and mitigates the severity of an attack, but it is the great germ-destroyer, and prevents the spread of this dread disease in households.

